# Impaired Glycine Receptor Trafficking in Neurological Diseases

**DOI:** 10.3389/fnmol.2018.00291

**Published:** 2018-08-21

**Authors:** Natascha Schaefer, Vera Roemer, Dieter Janzen, Carmen Villmann

**Affiliations:** Institute for Clinical Neurobiology, Julius-Maximilians-University Würzburg, Würzburg, Germany

**Keywords:** glycine receptor, startle disease, autoimmune antibodies, protein maturation, trafficking pathways

## Abstract

Ionotropic glycine receptors (GlyRs) enable fast synaptic neurotransmission in the adult spinal cord and brainstem. The inhibitory GlyR is a transmembrane glycine-gated chloride channel. The immature GlyR protein undergoes various processing steps, e.g., folding, assembly, and maturation while traveling from the endoplasmic reticulum to and through the Golgi apparatus, where post-translational modifications, e.g., glycosylation occur. The mature receptors are forward transported via microtubules to the cellular surface and inserted into neuronal membranes followed by synaptic clustering. The normal life cycle of a receptor protein includes further processes like internalization, recycling, and degradation. Defects in GlyR life cycle, e.g., impaired protein maturation and degradation have been demonstrated to underlie pathological mechanisms of various neurological diseases. The neurological disorder startle disease is caused by glycinergic dysfunction mainly due to missense mutations in genes encoding GlyR subunits (*GLRA1* and *GLRB*). *In vitro* studies have shown that most recessive forms of startle disease are associated with impaired receptor biogenesis. Another neurological disease with a phenotype similar to startle disease is a special form of stiff-person syndrome (SPS), which is most probably due to the development of GlyR autoantibodies. Binding of GlyR autoantibodies leads to enhanced receptor internalization. Here we focus on the normal life cycle of GlyRs concentrating on assembly and maturation, receptor trafficking, post-synaptic integration and clustering, and GlyR internalization/recycling/degradation. Furthermore, this review highlights findings on impairment of these processes under disease conditions such as disturbed neuronal ER-Golgi trafficking as the major pathomechanism for recessive forms of human startle disease. In SPS, enhanced receptor internalization upon autoantibody binding to the GlyR has been shown to underlie the human pathology. In addition, we discuss how the existing mouse models of startle disease increased our current knowledge of GlyR trafficking routes and function. This review further illuminates receptor trafficking of GlyR variants originally identified in startle disease patients and explains changes in the life cycle of GlyRs in patients with SPS with respect to structural and functional consequences at the receptor level.

## Introduction

Glycine receptors are pentameric ligand-gated ion channels that belong to the superfamily of Cys-loop receptors (CLRs). Other members of the CLR family are the nicotinic acetylcholine receptors (nAchR), GABA_A/C_ receptor and the 5HT_3_ receptor. Common to all CLRs is their pentameric arrangement of subunits around an ion channel pore as well as a conserved disulfide bridge in the large ECD.

The majority of post-synaptic GlyRs are formed by α1 and β subunits (Yang et al., 2012; Patrizio et al., 2017), and are anchored via binding of the GlyR β subunit to the anchoring protein gephyrin at the inhibitory post-synapse (Meyer et al., 1995). Gephyrin itself is bound to the actin cytoskeleton (Maas et al., 2006, 2009) and to polymerized tubulin (Kirsch et al., 1991). In addition, it is linked to the guanine nucleotide exchange factor collybistin (Kins et al., 2000), the post-synaptic adhesion molecule neuroligin-2 (Poulopoulos et al., 2009) and the actin-binding protein profilin (Mammoto et al., 1998). This protein network enables integration, rearrangement, and maturation of inhibitory synapses during development.

During maturation of GlyRs, receptor complexes fold and pass a network of cell compartments: the ER, the ER-Golgi intermediate compartment (ERGIC), *cis*-, *medial*-, *trans*-Golgi and the *trans*-Golgi-network (TGN). If these processes fail, and receptor proteins fold improperly, they remain within the ER. They will be translocated into the cytosol, followed by ubiquitination and subsequent degradation by the proteasome (Villmann et al., 2009b; Atak et al., 2015). This process is known as ER-associated degradation (ERAD).

Glycine receptor trafficking occurs via binding to the scaffold protein gephyrin, microtubule-associated motor proteins kinesin superfamily protein 5 (KIF5) (Maas et al., 2009), and dynein light chains 1 and 2 (dlc1/2) (Fuhrmann et al., 2002) in intracellular vesicles. This multi-protein complex is transported anterograde and retrograde between the neuronal cell body and distal neurites.

Glycine receptors are predominantly found in motoneuronal membranes of the adult spinal cord, brain stem, but also in the retina (Lynch, 2004). They enable Cl^-^ ion influx, generating hyperpolarization of the neuron. Disturbances in glycinergic inhibition at the functional level or impairment in the GlyR life cycle trigger neurological diseases such as startle disease (stiff-baby syndrome, hyperekplexia, OMIM 149400) (Harvey and Rigo, 2010) and are associated with pain mechanisms (Harvey et al., 2004b) as well as autism spectrum and panic disorders (Harvey and Yee, 2013; Pilorge et al., 2015; Deckert et al., 2017). Another rare neurological disorder associated with disturbed glycinergic inhibition is the GlyR autoantibody mediated form of stiff-person syndrome (SPS).

Startle disease is caused by mutated GlyRs or other proteins expressed at inhibitory synapses such as the pre-synaptic GlyT2, the post-synaptic scaffold proteins gephyrin or collybistin. Startle disease is characterized by high muscle tone, stiffness in infancy, and exaggerated startle reactions through sudden stimuli like noise or touch.

Mutations associated with startle disease are distributed across the overall GlyR sequence and have been identified in the GlyR α1 and β subunits (encoded by genes *GLRA1* and *GLRB*) (Bode and Lynch, 2014). They can be divided into dominant, recessive, or compound heterozygous inherited mutations. While amino acid exchanges leading to a dominant mutation mostly impair protein function, e.g., ligand binding, channel opening, or chloride gating, most recessive mutations exhibit defective cellular maturation and surface trafficking (Bode and Lynch, 2014).

SPS patients show a similar phenotype compared to patients suffering from startle disease including stiffness and painful spasms especially in axial and proximal limb muscles (Bhatti and Gazali, 2015; Dalmau et al., 2017). The most efficient treatment is immunotherapy but patient relapses are common (Hinson et al., 2018), which demonstrates that SPS is treatable but remediless. In contrast to mutations in GlyR genes underlying startle disease, SPS patients carry GlyR autoantibodies. The pathology of GlyR autoantibodies is not completely solved yet. It is proposed that autoantibody binding to the GlyR leads to receptor crosslinking and subsequent enhanced receptor internalization and degradation. As a consequence, the reduced GlyR numbers at the neuronal membrane generate impaired glycinergic neurotransmission.

In this review, we want to address mechanisms of GlyR biogenesis, e.g., GlyR assembly and maturation (I), receptor trafficking (II), and post-synaptic integration and clustering (III), GlyR internalization/recycling/degradation (IV). The second part will reflect disturbances of these processes based on findings for human and murine GlyR mutations associated with the neuromotor disorder startle disease. Furthermore, changes in GlyR degradation upon autoantibody binding to GlyRs as seen in SPS patients will be discussed.

## Molecular Insights Into Receptor Biogenesis and the Life Cycle of GlyRs

### Receptor Assembly and Maturation

**FIGURE 1:**
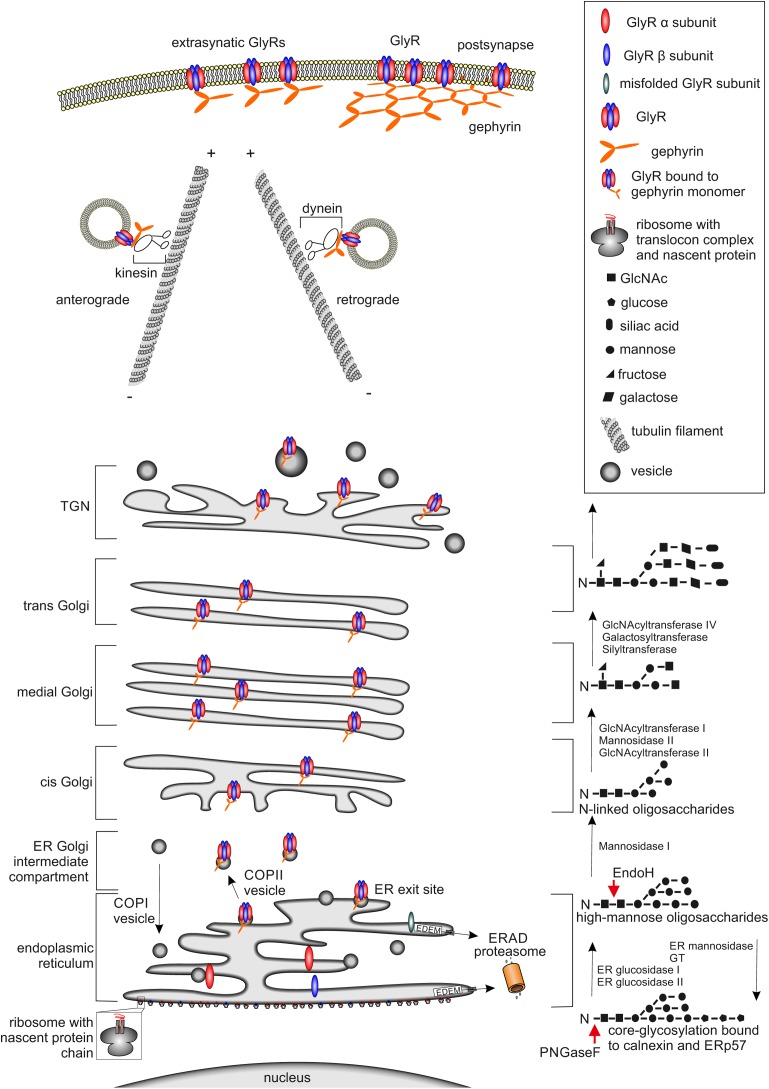
Glycine receptor maturation pathway. The GlyR maturation process is depicted. **(Left)** Schematic cell organelle-structures with important maturation proteins for the GlyRs are shown. **(Right)** Corresponding linked oligosaccharides to receptors, enzymes enabling glycosylation are labeled. The protein maturation starts with the establishment of single GlyR subunits single oval blue (β subunit) and red (α subunit) structures via translation at the ER. Within the ER, the assembly of single subunits to pentameric GlyR complexes occurs. GlyR complexes are then transferred to the *cis*-Golgi compartment attached to vesicles where further quality-control mechanisms take place. If GlyRs are misfolded, they will be recycled from the Golgi to the ER possibly by COPI-vesicles and degradation via mechanisms like ERAD leading to the proteasomal pathway occurs. Correctly folded and assembled GlyRs get glycosylated and transported through the secretory Golgi apparatus and *trans*-Golgi-network (TGN) and shuffled actively via kinesin-anterograde transport along tubulin filaments to the cell surface. The counterpart, retrograde transport of GlyRs is mediated by dynein instead of kinesin, which is responsible for the anterograde transport. At the cellular membrane the GlyR is associated to the scaffold protein gephyrin shown as orange hexagonal-lattice structure.

As a first step in general protein maturation, the nascent amino acid chain, which is established by the ribosome, enters the ER cotranslationally via the translocon complex (**Figure 1**, lower part). After the completed protein chain has been released from the ribosome through the translocon into the ER, post-translational folding and core-glycosylation takes place (Ellgaard and Helenius, 2003).

Post-translational glycosylation of proteins occurs by the addition of glycan structures to the growing and maturing amino acid strain. The glycosylation state of a protein is important for ER quality control and protein secretion. Although more than 7000 mammalian glycan structures exist, they are assembled from only 10 monosaccharides: fucose (fuc), galactose (Gal), glucose (Glc). *N*-Acetylgalactosamine (GalNAc), *N*-acetylglucosamine (GlcNAc), glucuronic acid (GlcA), iduronic acid (IdoA), mannose (man), sialic acid (SA), and xylose (Xyl) (Nairn et al., 2008).

Two different types of glycosylation are known: N-glycosylation and O-glycosylation. For N-glycosylation, the glycans are attached to asparagines embedded in a N-X-S/T motif (X can be any amino acid residue except proline or aspartate). *O*-Glycosylation occurs via the hydroxyl group of either serine or threonine residues (Kornfeld and Kornfeld, 1985). Within this glycan transfer, two GlcNAc, nine mannose residues, and three glucose molecules (Glc_3_Man_9_GlcNAc_2_) are transferred onto the growing protein at the luminal site of the ER (**Figure 1**, lower right part). Further protein maturation requires trimming of the first two glucose molecules to receive the formation GlcMan_9_GlcNAc_2_ as a ligand for calnexin or calreticulin which are important for ER quality control (Helenius and Aebi, 2004).

Glycine receptor protein maturation follows the above-mentioned pathways. N-Glycosylation of the GlyR takes place in the ER as a prerequisite of GlyR assembly and is necessary for GlyR ER exit (Griffon et al., 1999). Glycine receptors contain only one or two consensus sites for N-glycosylation. GlyR α subunits α1, α2, α3, and β carry a conserved N-glycosylation site (N-X-S/T) in the ECD (^38^NVS^40^ in α1, numbers refer to mature protein; ^45^NVT^47^ in α2, ^38^NVT^40^ in α3, and ^32^NST^34^ in GlyR β). GlyR β harbors an additional N-glycosylation site in the ECD (^220^NCT^222^ for GlyR β).

The glycosylation status of GlyRs can be estimated by digestion with the glycosidases Endo H and PNGase F. Endo H is able to remove high mannose type-glycans leaving only one *N*-acetylglucosamine attached at the asparagine (**Figure 1**) until later the Golgi α-glucosidase II cleaves off two mannose molecules. Removal of ^38^N from GlyR α1 led to ER accumulation of the protein. The introduction of another N-glycosylation site (introduction of an asparagine at position 33) led to a shift in the molecular weight upon Endo H treatment demonstrating that GlyR glycosylation cannot be prevented by mutation of ^38^N. Although glycosylation was possible, GlyR α1 lacking the consensus site for glycosylation was unable to exit the ER. Thus, residue ^38^N is crucial for ER exit of assembled GlyRs (Griffon et al., 1999).

As a further step of the ER maturation process oligomeric assemblies occur (Ellgaard and Helenius, 2003). GlyRs assemble in the ER as pentameric complexes. The assembly of GlyR α1 and β into homopentameric or heteropentameric receptors was revealed by site-directed mutagenesis. Several assembly boxes within the large ECD of GlyR α1 (^35^PPVNVSC^41^, ^74^AYNEYPDD^81^, ^90^LDSI^93^, ^125^NVLY^128^) were determined to promote oligomeric arrangement (Kuhse et al., 1993). Within the assembly boxes, eight residues namely ^38^N and ^40^S which form the putative N-glycosylation site and ^35^P, ^79^P, ^90^L, ^92^S, ^125^N, and ^128^Y were identified as crucial determinants for the different assembly properties of α and β subunits (Griffon et al., 1999). Surface labeling of injected *Xenopus* oocytes with radio-iodated substances and subsequent analysis on blue-native gels allowed the visualization of oligomeric and pentameric states (Kuhse et al., 1993; Griffon et al., 1999). In addition to the ECD assembly boxes, transmembrane domain 4 (TM4) as well as TM1 and TM3 were shown as essential domains required for GlyR pentamerization (Haeger et al., 2010). Haeger et al. (2010) used a mutagenesis approach splitting the GlyR α1 into an N- and C-terminal part. The N-terminal domain contained the ECD and TM1, 2, and 3, the C-terminal domain harbored most of the TM3-4 loop sequence, TM4 and the short C-terminus. Single expression of either the N- or the C-terminal domain resulted in aggregate formation or oligomerization but not pentamerization of GlyR domains. Coexpression of these two domains rescued GlyR pentamerization. A mutation series of all aromatic residues in the TM domains identified these residues within TM4 as essential determinants for pentamerization of GlyRs together with aromatic residues of TM1 and TM3. These aromatic residues form a ring structure between TM1, TM3, and TM4 enabling intrasubunit interactions between transmembrane segments most probably by π–π interactions. In addition to intersubunit interactions between GlyR ECDs, intrasubunit interactions between TMs have been suggested to play a significant role underlying pentamer formation of GlyRs (Haeger et al., 2010).

Similarly, truncated GlyR α1 variants lacking TM4, e.g., the truncated GlyR α1 variant from the *oscillator* mouse model, or truncated variants obtained originally from patients showed intracellular expression exclusively. However, upon coexpression with the lacking part containing the TM3-4 loop sequence, TM4, and the C-terminus surface expression of truncated GlyR protein was rescued. These data also support that the missing TM4 harbors essential determinants for pentamerization and finally ER export (Villmann et al., 2009a; Schaefer et al., 2015).

Chaperones, such as calnexin and calreticulin enable ER exit of proteins. Both are homologous proteins of a lectin family residing within the ER and able to bind only mono-glycosylated and N-linked core-glycans. Calnexin is a transmembrane protein, whereas calreticulin is a soluble luminal protein. Calnexin stays bound to the nascent protein until the remaining third glucose residue is removed (Helenius and Aebi, 2004). Hence, calnexin/calreticulin are responsible for ER quality control and prevent immature proteins from leaving the ER unfolded and unassembled.

Glycine receptor α1 proteins interact with calnexin as shown by coimmunoprecipitation studies (Schaefer et al., 2015). Mutated GlyR α1variants of the ECD loop β2-3 (W68C, D70N, and R72H) and TM4 variant W407R demonstrated increased protein–protein interactions with calnexin compared to α1 wild type (wild type refers to full-length non-mutated GlyR α1). In addition to enhanced coimmunoprecipitation with calnexin, these mutant GlyR α1 proteins revealed massive protein instability seen by a large fraction of protein degradation compared to wild type (Schaefer et al., 2015). For other CLRs protein interactions with chaperones have also been obtained, e.g., for AChRs where calnexin prolongs the lifetime of subunits approximately 10-fold, while association with BIP leads to a shortened lifetime (Wanamaker and Green, 2007).

Exit of proteins from the ER is enabled at transitional elements or ER exit sites (D’Alessio et al., 2010). Here, buds are formed or small membrane cluster that are contiguous with the ER membrane. The ER exit sites are coated with the coatomer protein II (COPII) (Barlowe, 2002) that will allow protein transfer to the Golgi apparatus. For the GlyR, protein interactions with COPII have not yet been investigated. Forward trafficking of the pre-synaptic GlyT2 is controlled by the COP II complex which allows transport from the ER to the *cis-*Golgi and through various Golgi cisternae (Arribas-Gonzalez et al., 2015). In contrast to COPII, COP I is responsible for retrograde trafficking of proteins from the Golgi apparatus back to the ER for degradation (Popoff et al., 2011). Binding of the GlyR or GlyT2 to COPI has not been shown yet.

From other CLRs it is known, e.g., that the biogenesis of nAchR is controlled by the ER protein RER-1 which allows forward trafficking of pentameric ion channels only (Valkova et al., 2011). For GABA_A_ receptors it is described that only certain combinations of subunits are transported from the ER to the Golgi, however, the quality control mechanism that enables only functional receptor complexes to reach the cellular surface is not understood yet (Kittler et al., 2002). GlyRs as well as GABA_A_ receptors that are ready to leave the ER are not only assembled as heteropentamers but also coupled to gephyrin before leaving the ER (Hanus et al., 2004). The association between the GlyR pentamer and gephyrin promotes the accumulation of GlyRs at the cellular surface (Hanus et al., 2004).

The ER is continuously feeding cargo-containing vesicles into the secretory pathway and receiving retrograde membrane traffic from the Golgi complex in return. Resident ER proteins, including chaperones and folding enzymes, contain ER-retention and ER-retrieval signals and stay in the ER (Harter and Wieland, 1996; Teasdale and Jackson, 1996). The tetrapeptide KDEL sequence is present at the carboxyl-terminus of many soluble ER proteins (**Figure 1**) (Munro and Pelham, 1987; Pelham, 2000). KKXX or KXKXX signals and exposed cysteine residues can also function as retention signals in the lumen of the ER (Fra et al., 1993; Pelham, 1994). Retention and retrieval signals play an important role within the quality control of proteins destined for export. The best caracterized signal is the RKR motif. This RKR motif helps to retain individual subunits and incomplete oligomers in the ER until they are masked by the correct oligomeric assembly (Ma and Jan, 2002).

Similar basic motifs have also been detected in the TM3-4 loop of the GlyR α subunits. The intracellular TM3-4 loop of GlyR α1 harbors such a basic domain ^318^RRKRRH^323^ at its N-terminal part which is also present in other GlyR α subunits (partially present in α2 ^325^RRRQKR^330^ and α3 ^318^RRKRKN^323^). This motif is most probably masked upon oligomeric GlyR assembly and forward trafficking of GlyR oligomers to the cellular surface is enabled (Sadtler et al., 2003).

If the GlyR α1 is truncated C-terminal to ^318^RRKRRH^323^, this truncated α1 variant is still able to oligomerize and exit the ER but no functional GlyR pentamers are formed (Villmann et al., 2009a; Haeger et al., 2010). As has been pointed out before, when the lacking C-terminal domain is coexpressed with truncated GlyR α1, correctly pentamerized GlyR are transported to the cell surface that form functional chloride channels (Villmann et al., 2009a; Haeger et al., 2010). These data further support that TM4 is an essential regulator of GlyR pentamerization but not oligomerization.

Proteins that will later be exposed on the cellular or neuronal surface undergo a second step of quality control. A special form of proteins that escort surface proteins from the ER to the Golgi, e.g., receptor associated protein (RAP), bind to members of the low density lipoprotein-receptor and prevent premature ligand-binding in the early secretory pathway (Bu, 2001). Besides RAP, ERGic-53 is also known to cycle between ER and Golgi and transport high mannose N-linked glycans to the Golgi apparatus (Ellgaard and Helenius, 2003). The GlyR also interacts with ERGic-53 during ER-Golgi transport. This interaction was demonstrated for GlyR α1 wild type. First hints for disrupted ER-Golgi trafficking and finally disturbed GlyR biogenesis as a pathomechanism in human startle disease was given by lack of interaction between cycling protein ERGic-53 and GlyR α1 mutants identified from human patients (Schaefer et al., 2015). Further studies are required to identify other interaction proteins of the GlyR either in the ER or ERGIC involved in feed forward or feed back mechanisms during GlyR biogenesis.

### Receptor Trafficking

After leaving the ER, glycoproteins traverse to the Golgi apparatus, where attached glycans are susceptible to further processing (**Figure 1**). To date there are still different models for Golgi protein trafficking (e.g., cisternal maturation, vesicular transport, and rapid partitioning), where each model can explain different observations in protein transport (Glick and Luini, 2011; Moremen et al., 2012). Cisternal maturation is one of the earliest models for Golgi trafficking and proposes that cisternae are formed by fusion and maturation of vesicles leaving the ER. This model has its origin in electron microscopy observations of Golgi morphology (Mironov et al., 2001).

Within the maturation pathway, almost all glycoprotein glycans are subject to trimming and extension via glycosyltransferases. *Cis-*Golgi compartments contain enzymes that initiate O-linked glycosylation and mannosidases for trimming high mannose N-linked glycans. Medial compartments contain enzymes that branch O-linked glycans. Furthermore, complex N-linked glycan formation and branching is initiated (Roth, 2002; Tu and Banfield, 2010). The N-linked glycans on glycoproteins become resistant to removal by Endo H (**Figure 1**, right). Endo H-resistant glycan structures are thus utilized to verify protein translocation to the Golgi apparatus (Rothman and Fine, 1980; Moremen et al., 2012).

Endo H resistance has been shown for mature GlyR complexes. The resistance to Endo H is due to additional glycosylation modification (Bormann et al., 1993). Another enzyme used to determine the glycosylation status of a protein is PNGase F, which cuts mannose-chains directly attached at asparagine residues. Using PNGase F, it was identified that the glycosylation status of some GlyR α1 mutants originally identified in patients with startle disease differed from GlyR α1 wild type. The GlyR α1 mutants received only core glycosylation enabling trafficking toward ERGIC but preventing transport to the Golgi secretory pathways. Hence, impaired GlyR assembly and trafficking are pathomechanisms underlying startle disease (Schaefer et al., 2015).

These proteinbiochemical data were accompanied by an analysis of the trafficking routes using compartmental markers for ER, ERGIC, and *cis-*Golgi (Schaefer et al., 2015). Several GlyR α1 mutants revealed differences in the passage through compartments like ER, ERGIC, and *cis*-Golgi, and showed aggregate accumulation in the ER (Schaefer et al., 2015). These data suggest different mechanisms underlying disease pathology: (i) the mutations lead to misfolding as primary source for ER accumulation, (ii) the mutations do not cause dramatic structural changes, which are recognized by the quality control, (iii) the ER quality control system might be leaky and allows forward trafficking. Very fast protein turnover may also underlie such observations.

*Trans-*Golgi compartments elaborate additional branching and capping reactions (galactosylation, sialylation, sulfation, and external fucosylation) on complex N-linked and O-linked antennae (Stanley, 2011). Beyond the *trans-*Golgi, capping reactions are continued in the *trans-*Golgi network (TGN), a tubulovesicular compartment that stages glycoprotein cargos for secretion or sorting to specific cell surfaces or subcellular membrane domains via transport by Kif5/dynein and microtubule filaments (Maas et al., 2006; Reynders et al., 2011; Moremen et al., 2012) (**Figure 1**, right).

Exit of the GlyR from the TGN was investigated using incubation at various temperatures to either block GlyRs from TGN exit or allow GlyR release from the TGN followed by temporal analysis of receptor incorporation into the surface membrane of neurons (Hanus et al., 2004). It was shown that binding of gephyrin to the GlyR modifies cellular distribution of GlyRs and determines the speed of GlyR movement in the cell. The radial and directed movements of GlyR-gephyrin complexes to the PM suggested a vesicle associated transport in the cytoplasm. Furthermore, the diffusion coefficient of the cytoplasmic GlyR-gephyrin complexes was comparable with that of vesicle tracking along microtubules (Hanus et al., 2004). Following exit from the Golgi apparatus, the anterograde transport of GlyR-gephyrin complexes is enabled by the motor protein kinesin (KIF5) along microtubules to reach the neuronal cell surface, while retrograde transport to intracellular compartments is appoved by complexes coupled to dynein (Fuhrmann et al., 2002; Kneussel, 2005; Maas et al., 2009).

### Post-synaptic Integration and Clustering

Once the receptor reaches the cellular membrane it is inserted in correct topology. The intracellular TM3-4 loop of GlyR α1 harbors a basic domain ^318^RRKRRH^323^, which contains the ER retention motif RKR. This motif is masked by correct GlyR oligomerization and allows GlyR protein exit from the ER. The basic domain ^318^RRKRRH^323^, however, is a multifunctional motif in the GlyR and also important for sufficient membrane integration (Sadtler et al., 2003). When two of the positively charged residues in ^318^RRKRRH^323^ are mutated in the GlyR, surface integration is diminished. Consequences of a disruption of the basic motif were also investigated using Endo H and PNGase F digestion. Indeed the mutated GlyRs were cleavable by Endo H arguing that the GlyR α1 protein lacking some basic residues at the intracellular site of TM3 will be retained in the ER (Sadtler et al., 2003).

The transport of the GlyR-gephyrin complex along microtubules associated with Kif5 is a prerequisite for post-synaptic integration and GlyR clustering. A disruption of the GlyR-gephyrin complex prevents formation of GlyR clusters at the inhibitory synapse (Kirsch et al., 1993; Zacchi et al., 2008), a mechanism that is also obvious in gephyrin-deficient mice (Feng et al., 1998). Integration into the inhibitory synapse requires guidance by other proteins, e.g., collybistin. Collybistin represents an integrating regulatory node within the formation and function of inhibitory post-synapses (Soykan et al., 2014). Thus, it is not surprising that a mutation in collybistin (G55A) causes mislocation of gephyrin and the associated GABA_A_Rs and GlyRs (Harvey et al., 2004a). The surface transport of GlyR-gephyrin complexes is, however, independent from collybistin (Papadopoulos and Soykan, 2011).

Several studies showed the high dynamic mobility of GlyRs in the cellular membrane. GlyRs change fast between extrasynaptic, perisynaptic, and synaptic sites which allows quick adaptation to changes in synaptic activity and finally synaptic plasticity. Exocytosis of newly synthesized receptors appears at non-synaptic sites at the cell body and initial portions of dendrites (Rosenberg et al., 2001). Surface integrated receptors exist as discrete clusters which are stable in size. These clusters appear in distal dendritic regions and result from receptor diffusion and not from exocytosis of transported vesicles directed to distal dendrites (Rosenberg et al., 2001). Using the single particle tracking method it became possible to follow the surface movements of GlyRs in real time. If the used single particle is smaller than 1 μm, movements of a particle bound receptor are similar to those of an individual receptor (Kucik et al., 1999). The mobility of GlyRs depends on the interaction with gephyrin. GlyR expression without gephyrin result in freely diffusing receptors whereas the association with gephyrin induces long confinement periods spatially associated with submembranous clusters of gephyrin (Meier et al., 2001). In a follow-up study, quantum dots (QDs) which are nanometer-sized probes with a long-lasting fluorescence emission brought a breakthrough in determination of receptor dynamics at neuronal surfaces. Fast exchanges of GlyRs between extrasynaptic und synaptic compartments became quantitatively analyzable by trajectory analysis. QD tracked receptors have never been found intracellularly. The analysis of the receptor trajectories was coupled to electron microscopy to precisely localize the diffusing GlyRs. These images provided the most direct evidence that QD-coupled GlyRs are present in the core of synapses (Dahan et al., 2003). This technique was also used to determine the homeostatic regulation of receptor numbers at synapses by ongoing neuronal activity. In the spinal cord, most inhibitory synapses have a mixed glycine-GABA phenotype. It was shown that excitation influences the mobility of GlyR receptors but not of GABA_A_ receptors. Changes in neuronal activity very rapidly lead to a change in GlyR lateral diffusion. Normally an equilibrium exists between a functional receptor pool at synapses and a reserve pool at extrasynaptic sites (Triller and Choquet, 2005). Receptor entry in and exit from synapses is balanced by neuronal activity and finally determines the number of GlyRs at synapses. The homeostatic regulation of receptors at synapses is slow and takes place in the order of hours to days (Turrigiano and Nelson, 1998). Thus, changes in diffusion properties precede changes in receptor numbers at synapses (Levi et al., 2008). Recent advances of single molecule imaging allowed the very precise structural characteristics of synapses, high-resolution cartography of the diffusive behavior of ligand-gated ion channels such as the GlyR, quantification of synaptic components and trapping energies between receptors and the scaffold proteins such as gephyrin (Salvatico et al., 2015). The development of a combination of such technically ambitious techniques with theoretical approaches provide future perspectives to compare and differentiate physiological and pathological changes of synapse stability.

Besides trafficking and synaptic integration, surface expressed GlyRs have been analyzed for their stoichiometry using different approaches ranging from expression of defined GlyR subunit ratios, analysis of α1β concatemers, imaging of single antibody-bound α1β receptors using atomic force microscopy, and quantitative photoactivated localization microscopy (Grudzinska et al., 2005; Durisic et al., 2012; Yang et al., 2012; Patrizio et al., 2017). Two configurations, 3α:2β and 2α:3β have been proposed. Recent data on spinal cord neurons using quantitative photoactivated localization microscopy determined a stoichiometry of 3α:2β for α1-containing and α3-containing receptors. Moreover, α1-containing receptors were more mobile than α3-containing receptors and cluster with a lower density in synapses. The differential regulation of the clustering of α1- and α3-containing receptors may play a role in neuron-specific changes of glycinergic inhibition during inflammation (Patrizio et al., 2017).

### GlyR Recycling/Degradation/Internalization

**FIGURE 2:**
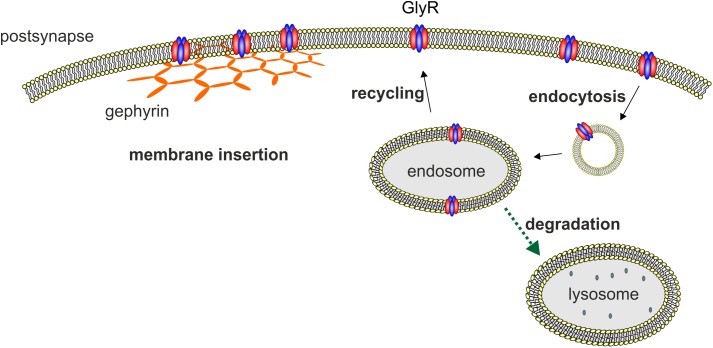
Membrane insertion, endocytosis, recycling, and degradation of the GlyR. GlyRs are associated to the hexagonal lattice of the scaffold protein gephyrin when membrane inserted. GlyR degradation occurs by endocytosis as a consequence of ubiquitination followed by lysosomal or lysosomal-like vacuolar degradation. From the endosome, GlyRs can also be recycled and transported back to the cellular surface and transported back to the cellular membrane.

Glycine receptor degradation at synaptic sites regulates the surface receptor numbers and thus influences synaptic plasticity. For GlyR degradation two independent mechanisms have been demonstrated at different stages of the GlyR life cycle. GlyR homeostasis in the normal GlyR life cycle is characterized by a defined turnover rate and either recycling or lysosomal degradation after receptor endocytosis (**Figure 2**). These processes differ from GlyR degradation as a consequence of GlyR misfolding and proteasomal degradation by the ERAD pathway. A third mechanism might exist following enhanced GlyR internalization as a consequence of autoantibody crosslinking of receptor proteins. So far, upon autoantibody binding GlyR translocation to the early endosomal compartment has been shown suggesting that further GlyR processing might follow the lysosomal degradation pathway (**Figure 2**, Carvajal-Gonzalez et al., 2014).

Depending on the cell system used, different half-life times of GlyRs have been reported. The half-life of GlyRs is between 14 h for a biotinylated GlyR and 2 days for the membrane spanning 49 kDa subunit (Hoch et al., 1989; Rasmussen et al., 2002). Another study showed a half-life of wild type GlyR α1 in transfected HEK293 cells of about 24 h (Villmann et al., 2009b). Buttner et al. (2001) demonstrated GlyR internalization and degradation is preceded by ubiquitination. Moreover, GlyRs are proteolytically nicked in the endocytic pathway into fragments of 35 and 13 kDa and are degraded mainly by the lysosomal pathway. In contrast, improperly folded GlyR proteins that do not reach the cellular surface and remain in the ER are translocated to the cytosol for ubiquitination and subsequent degradation by the proteasome (ERAD). Using lactacystin as a blocker of the proteasome, it was shown that ER accumulated GlyR mutants are degraded by the proteasomal pathway and not by the lysosomal pathway as observed for GlyRs that reached the cellular surface (Buttner et al., 2001; Villmann et al., 2009b; Schaefer et al., 2015).

## Impaired GlyR Maturation and Trafficking Associated With Human Startle Disease

The pathology of human startle disease is associated with GlyR maturation and trafficking defects. In particular, recessive GlyR mutations in either the *GLRA1* or *GLRB* gene encoding for subunits α1 and β impair GlyR biogenesis (Vergouwe et al., 1999; Rea et al., 2002; Villmann et al., 2009b; Chung et al., 2010). Moreover, these mutants serve as excellent tools to understand at which steps during protein maturation GlyRs stop trafficking and accumulate or get conveyed to degradation pathways of the cell.

**FIGURE 3:**
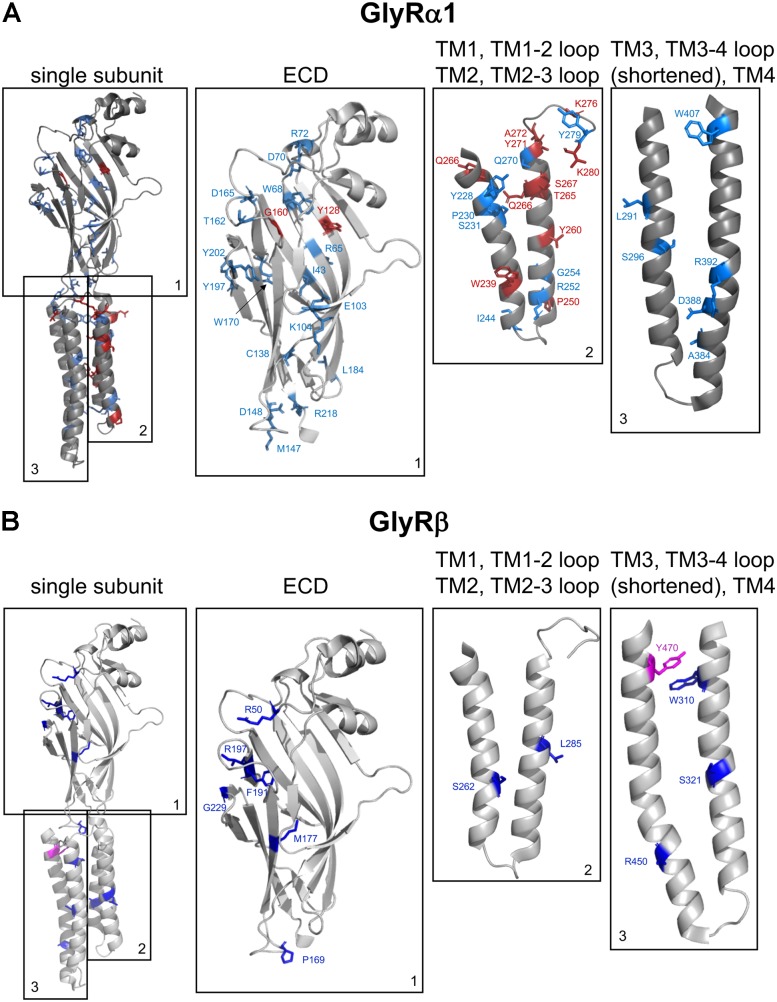
Structure of a single GlyR subunit including mutations associated with startle disease. Side view onto the principle subunit of GlyR α1 and β based on GlyR α3 (5VDH, Huang et al., 2015). **(A)** GlyR α1 subunit with marked residues by numbers of the amino acids affected by recessive (blue) and dominant (red) mutations (see also **Table 1**), parts of the GlyR, e.g., ECD (1); TM1, TM1-2 loop, TM2, and TM2-3 loop (2); TM3, TM3-4 loop (shortened according to Huang et al., 2015) and TM4 (3). **(B)** Single GlyR β subunit with residues labeled that have been identified as recessive (dark-blue) and dominant (pink) mutations (see also **Table 2**). The following mutations are not shown due to lack of structural information: residues within the TM3-4 loop – GlyR α1 R316X, G342S and G3475X and GlyR β E24X. Images were made using The PyMOL Molecular Graphics System, Version 1.8 Schrödinger, LLC.

There is no distinct structural region within the GlyR sequences where recessive GlyR mutations are mainly localized. Rather they are distributed across the whole GlyR α1 and β sequences (**Figure 3**). Several independent groups have shown *in vitro* that recessive GlyR α1 or β mutations lead to a reduction of surface expressed receptors. In turn, reduced receptor numbers at the cellular surface result in decreased glycine efficacy or non-functionality of the remaining GlyRs. The observed reduction of GlyRs at the cellular membrane implies that mutant receptors get lost on their track to the cellular surface. These observations raised several questions: Are mutant receptors lost due to an accumulation of misfolded receptor in the ER compartment? Are mutated GlyRs unable to exit the ER, unable to pass the ER-Golgi intermediate compartment and the Golgi apparatus to finally traffic to the cellular surface? When structural changes are tolerated, and ER exit is allowed, do these mutated receptors show higher protein turnover rates or changes in protein stability?

**Table 1:** Mutations in *GLRA1* encoding the GlyR α1 subunit and functional consequences.

Mutation	GlyRα1					Defect		
				
	GlyR subunit	Compound heterozygous	Inheritance	Location in GlyR		Biogenesis	Function	Reference
del ex1-7			Recessive	ECD		No protein expression		Brune et al., 1996; Becker et al., 2006
del ex4-7		R65L	Recessive	ECD		No protein expression		Chung et al., 2010
I43F			Recessive	ECD	β1		Gain-of-function	Horváth et al., 2014; Zhang et al., 2016
R65W/L		P230S, del ex4-7	Recessive	ECD	β2	Trafficking	Non-functional	Chung et al., 2010
W68C		R316X	Recessive	ECD	β2	Trafficking	Non-functional	Tsai et al., 2004; Schaefer et al., 2015
D70N		W407N	Recessive	ECD	β2	Trafficking	Non-functional	Schaefer et al., 2015
R72fsX47			Recessive	ECD	β2-β3			Rees et al., 2001
R72H/C			Recessive	ECD	β2-β3	Trafficking	Non-functional	Coto et al., 2005; Bode et al., 2013; Schaefer et al., 2015
E103K		L184fs21X	Recessive	ECD	β5		Functional	Chung et al., 2010
K104fsX47			Recessive	ECD	β5			Zoons et al., 2012
Y128C			*Dominant*	ECD	β6			Chung et al., 2010
C138S		D148fsX16	Recessive	ECD	β6-β7 (Cys loop)			Chan et al., 2014
M147V			Recessive	ECD	β6-β7 (Cys loop)			Rees et al., 2001
D148fsX16		C138S	Recessive	ECD	β6-β7 (Cys loop)			Chan et al., 2014
G160R			*Dominant*	ECD	β7-β8		Change in glycine EC_50_	Schaefer et al., 2015
T162M			Recessive	ECD	β7-β8	Trafficking	Change in glycine EC_50_	Schaefer et al., 2015
D165G			Recessive	ECD	β7-β8	Trafficking		Chung et al., 2010
W170S			Recessive	ECD	β8		Impaired zinc inhibition	Al-Futaisi et al., 2012; Zhou et al., 2013; Zhang et al., 2016
L184fs21X		E103K	Recessive	ECD	β8-β9			Chung et al., 2010
Y197X		Y202X	Recessive	ECD	β9			Chung et al., 2010
Y202X		Y197X	Recessive	ECD	β9			Rees et al., 2001
R218W/Q		S296X	Recessive	ECD	β10	Trafficking		del Giudice et al., 2001; Bode et al., 2013
Q226E			*Dominant*	TM1			Change in glycine EC_50_	Bode et al., 2013
Y228C			Recessive	TM1				Forsyth et al., 2007
P230S		R65W	Recessive	TM1			Change in glycine EC_50_	Bode et al., 2013
S231N/R		S296X	Recessive	TM1		50% surface expression, less stable than wild type	Low I_max_, change in glycine EC_50_	Humeny et al., 2002; Chung et al., 2010
W239C			*Dominant*	TM1				Gilbert et al., 2004
I244N			Recessive	TM1		Less stable than wild type	Low I_max_	Rees et al., 2001; Villmann et al., 2009b
P250T			*Dominant*	TM1-2			Fast desensitization	Saul et al., 1999; Breitinger et al., 2001; Breitinger and Becker, 2002
R252C/H/G		R392H	Recessive	TM2		Trafficking	Non-functional	Vergouwe et al., 1999; Villmann et al., 2009b
G254D			Recessive	TM2		Trafficking	Non-functional	Chung et al., 2010
V260M			*dominant*	TM2		Slightly reduced surface expression	change in glycine EC_50_, reduced sensitivity to partia agonists	del Giudice et al., 2001; Castaldo et al., 2004
T265I			*Dominant*	TM2			Increase in glycine EC_50_	Chung et al., 2010
Q266H			*Dominant*	TM2			Functional, increase in glycine EC_50_	Milani et al., 1996; Castaldo et al., 2004
S267N			*Dominant*	TM2			Functional, low ethanol sensitivity	Becker et al., 2008
S270T			*Dominant*	TM2				Lapunzina et al., 2003
R271Q/L/P/X			*Dominant*	TM2			Functional, reduced glycine sensitivity and single-channel conductance	Shiang et al., 1993; Langosch et al., 1994; Rajendra et al., 1995; Gregory et al., 2008; Lee et al., 2013; Bode et al., 2013
A272P			*Dominant*	TM2				Mine et al., 2015
K276E/Q			*Dominant/K276Q de novo*	TM2-3			Reduced glycine sensitivity, reduced open probability	Shiang et al., 1995; Elmslie et al., 1996; Lynch et al., 1997; Lewis et al., 1998; Mine et al., 2015
Y279C/S/X			*Dominant/Y279X Recessive*	TM2-3			Reduced glycine sensitivity, reduced whole cell current magnitude	Lynch et al., 1997; Gilbert et al., 2004; Poon et al., 2006; Mine et al., 2015
V280M			*Dominant*	TM2-3			Change in glycine EC_50_	Bode et al., 2013
L291P		D388A	Recessive	TM3		Trafficking	Change in glycine EC_50_	Bode et al., 2013
S296X		S231N and R218Q	Recessive	TM3		Reduced expression	Non-functional, reduced I_max_ with α1 wildtype	Bellini et al., 2007; Chung et al., 2010
R316X		W68C, R392H	Recessive	TM3-4		Trafficking	Non-functional	Tsai et al., 2004; Mine et al., 2015; Schaefer et al., 2015
G342S			Recessive	TM3-4			Functional	Jungbluth et al., 2000; Rees et al., 2001
E375X			Recessive	TM3-4		Trafficking		Bode et al., 2013
A384P		R392H	Recessive	TM3-4			Functional, desensitization impaired	Wang et al., 2018
D388A		L291P	Recessive	TM3-4		Trafficking	Change in glycine EC_50_	Bode et al., 2013
R392H		R252H, A384P, R316X	Recessive	TM4		Trafficking	functional	Villmann et al., 2009b; Chung et al., 2010; Mine et al., 2015
W407R			Recessive	TM4		Trafficking	Non-functional	Schaefer et al., 2015
R414H			*Dominant*	TM4			Functional	Bode et al., 2013
protein								


*Amino acid residues refer to mature.*

**Table 2:** Mutations in *GLRB* encoding the GlyR β subunit and functional consequences.

GlyRβ1					Defect		
			
GlyR subunit	Compound Heterozygous	Inheritance	Location in GlyR		Biogenesis	Function	Reference
F-191fsX3		Recessive	ECD				Chung et al., 2013
Δexl-8		Recessive	ECD				Chung et al., 2013
Δex5	G229D	Recessive	ECD				Rees et al., 2002
Δex5 and		Recessive	ECD				Chung et al., 2013
S176RfsX6							
E24X		Recessive	ECD	N-terminus			Chung et al., 2013
R50X	Q216fsX222	Recessive	ECD	α1-β1			Mine et al., 2013
P169L		Recessive	ECD	β6-β7 (Cys loop)	Reduced surface expression	Functional	Chung et al., 2013
M177R		Recessive	ECD	β7			Al-Owain et al., 2012
R190X	ΔS262	Recessive	ECD	β8		Functional	Chung et al., 2013
Q216fsX222	R50X	Recessive	ECD				Mine et al., 2013
G229D	Δex5	Recessive	ECD	β10			Rees et al., 2002
ΔS262	R190X	Recessive	TM1		Reduced surface expression	Functional	Chung et al., 2013
L285R		Recessive	TM2		Reduced surface expression		James et al., 2013
W310C		Recessive	TM2-3		Reduced surface expression		James et al., 2013
S321F	In4 (c.298-1G < A)	Recessive	TM3				Lee et al., 2013
R450X		Recessive	TM3-4		Reduced surface expression	Functional	Chung et al., 2013
Y470C		*Dominant*	TM4		Reduced surface expression	Functional	Chung et al., 2013


*Amino acid residues refer to mature protein.*

In the following section, we will discuss the known mutated GlyR variants with respect to protein biogenesis and functional deficits according to their location in the GlyR protein sequence – ECD, transmembrane domain (TMD), and ICD.

### Mutations in the GlyR α1 ECD Associated With Hyperkplexia

The GlyR ECD is composed of a short α-helical domain followed by 10 β-sheets connected by loop structures (Du et al., 2015; Huang et al., 2015). With two exceptions (Y128C and G160R), all mutations in the GlyR α1 ECD are recessively inherited. Recessive GlyR α1 mutants were identified in extracellular β-sheets β1, β2, β5, β6, β8, β9, β10 and within loops β2–β3 (loop D), loop β6–β7 (Cys loop), β7–β8 (loop B), and β8–β9 (loop F). Reduced surface expression (R65W, W68C, D70N, R72C, E103K, D165G, R218W) has been used as a quick readout for impaired GlyR biogenesis (Chung et al., 2010; Bode et al., 2013; Schaefer et al., 2015).

**FIGURE 4:**
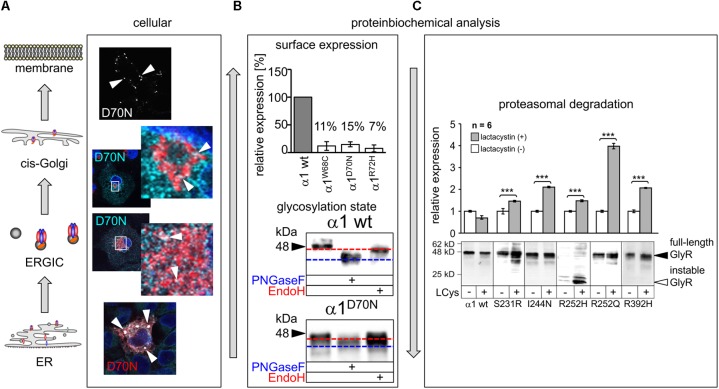
Biogenesis and trafficking defects of GlyR α1 mutants. **(A)** GlyR α1 subunits carrying recessive mutations are associated with trafficking defects to the cellular surface. Compartmental analysis of transfected COS7 cells with the GlyR α1 mutant α1^D70N^ showed very few protein dots (marked by white arrow heads) at the cellular surface (labeled before fixation with the α1 specific antibody mAb2b, 1:500). GlyR α1 protein analysis on the ER-ERGIC-*cis*-Golgi trafficking route determined GlyR α1^D70N^ staining in all compartments analyzed (for ER – calnexin cyan, GlyR α1 red; ERGIC – ERGic53 red, GlyR α1 cyan; *cis*-Golgi – GM130 red, GlyR α1 cyan). GlyR α1^D70N^ protein accumulation was most pronounced in the ER (large white dots). **(B)** GlyR protein glycosylation is a pre-requisite for ER exit. The status of protein glycosylation can be determined by digestion with glycosidases Endo H and PNGase F. PNGase F removes all N-linked oligosaccharides from glycoproteins (blue dotted line, lower images). Endo H cuts within the core of high mannose and some hybrid oligosaccharides from N-linked glycoproteins. Once a protein enters the Golgi apparatus and is further glycosylated, the protein gets resistant to Endo H digestion (red dotted line, lower images). A comparison of surface expression between GlyR wild type and mutants can be achieved by biotinylation of surface receptor protein and subsequent binding and elution from streptavidin-beads (upper image). The protein analysis is done normalized to a house keeping protein (e.g., pan-cadherin) and the assumption that the GlyR α1 wild type expression refers to 100%. **(C)** GlyR α1 mutants are degraded by proteasomal degradation. Using lactacystin (LCys), a proteasomal blocker, GlyR α1 mutant proteins accumulated significantly. The quantification showed significant protein increase following lactacystin treatment for all GlyR α1 mutants but not for the wild type. Images in **(A–C)** are were modified from Villmann et al. (2009b) and Schaefer et al. (2015). ^∗∗∗^*p* < 0.001.

More detailed analyses are scarce. Mutations in β2 and the β2-β3 loop (R65W, W68C, D70N, R72H) showed dramatic decrease of surface receptors (7–15% compared to the wild type GlyR α1, **Figure 4**). Only very few receptor signals have been found in immunocytochemical stainings of living cells (Schaefer et al., 2015). Some of the characterized β2 and β2-3 loop mutations were identified in patients carrying an additional mutation somewhere else in the *GLRA1* gene generating compound heterozygosity, such as W68/R316X, and D70N/W407R. Independent of the origin of these mutations, all β2 and β2-3 loop GlyR α1 variants, when expressed recombinantly in HEK293 cells, resulted in non-functional channels arguing that a surface expression of less than 15% is not sufficient to result in functional ion channels.

The mutation T162M in loop β7–β8 of the GlyR α1 led to 53% of surface receptors. 53% receptor protein at the surface is sufficient to generate functional GlyRs but results in significantly reduced maximal currents and reduced glycine potency (Schaefer et al., 2015). Hence, receptor expression is differently affected by mutations in different regions of the GlyR protein. In addition to changes in the number of surface receptors and reduced glycine efficacy and potency, an increased propensity for aggregation of T162M monomers was identified (Schaefer et al., 2015). The increased aggregation of T162M receptors did not disable a feed-forward transport of these mutated receptors from the ER to the cellular membrane. Using high-resolution confocal microscopy, large ER accumulations of mutated GlyRs (T162M but also W68C, D70N, R72H) were obvious colocalizing with the ER marker calreticulin. These large ER accumulations did not prevent the mutated receptors to exit the ER and enter the ER-Golgi intermediate compartment (ERGIC) as well as *cis-*Golgi. Receptor transport was further traced with colocalization analysis using marker proteins for the ER-Golgi intermediate compartment (ERGic53) and *cis-*Golgi GM130. These subcellular compartments are recognized by their donut-like staining pattern in the cytoplasm of cells (**Figure 4A**). The passage of the Golgi compartment was further confirmed by the comparison of the glycosylated/unglycosylated expression pattern (**Figure 4B**). In neurons, subcompartmental analysis of the GlyR β2 and β2-β3 loop mutants revealed similar results to transfected cell lines arguing that the neuronal trafficking is impaired in some forms of hyperekplexia (Atak et al., 2015; Schaefer et al., 2015).

A reduction in the surface expression does, however, not necessarily mean that the receptor is non-functional. In contrast to non-functional homomeric α1 variants R65W, W68C, D70N, R72C, and R218W, mutations Y128C and D165G exhibited residual functionality in both homomeric and heteromeric receptor configurations (Bode and Lynch, 2013). Moreover, glycine application to HEK293 cells transfected with E103K resulted in large maximal current amplitudes indistinguishable from wild type (Chung et al., 2010). When mutants R65W, R72C, and R218W were coexpressed with wild type α1, functional receptors were observed with different physiological properties arguing for heteromeric assemblies of mutated and non-mutated subunits. Coexpression with either wild type α1 or α1β is used to simulate the *in vivo* receptor configuration if we assume a heteromeric receptor stoichiometry of 3α:2β. Common to all recessive mutations independent of residual functionality or functional ion channel formation in coexpressions of mutated subunits with wild type α1 (or α1β) is their impaired glycine potency. Glycine potency is significantly reduced, e.g., glycine EC_50_ values increase threefold for R72C + wild type α1, and 25-fold for E103K (Chung et al., 2010; Bode and Lynch, 2013). Reduced glycine potencies by 3–25 fold suggest that a higher concentration of the neurotransmitter glycine is required for half-maximal activation of the GlyR channels. Compared to these *in vitro* results, synaptic glycine concentration can reach up to 5 mM during synaptic activity. Thus, the observed values are in line with the non-symptomatic phenotype of patients carrying recessive GlyR mutations. In the closely related 5HT_3_ receptor, electrophysiological characterization of chimeric mutants and mutants carrying point mutations in the ECD also demonstrate the contribution of ECD (loops β9-β10 and β8-β9) to curare potency (Zhang et al., 2007).

The previously identified mutation W170S localized in β-sheet β8 has been demonstrated as a gain-of function mutation. Accelerated decay and rise times were observed for this mutant (Zhang et al., 2016). Additionally, W170 is an important binding residue for Zn^2+^-mediated GlyR potentiation (Zhou et al., 2013).

Recently, missense mutations within the *GLRB* gene have been identified. The GlyR β ECD carries only recessive mutations (P169L, M177R, and R190X, **Figure 3**) (Chung et al., 2013; James et al., 2013). Mutants P169L and R190X exhibit trafficking defects and show significantly reduced surface expression levels (Chung et al., 2013). The remaining receptor population at the cellular surface resulted in heteromeric receptors of mutated GlyR β subunits with non-mutated α1 subunits with almost no change in glycine sensitivity but reduced chloride ion influx (Chung et al., 2013). A 50% reduction in maximal currents amplitudes observed for mutant M177R cannot be explained by reduced surface expression, since the M177R expression pattern is indistinguishable from GlyR β wild type (James et al., 2013). These data show that the reduction in maximal current amplitudes is not concomitant to reduced receptor levels at the cellular membrane and vice versa. Reduced I_max_ might also be due to structural changes at subunit interfaces in the heteromeric receptor configuration being transduced to the ion channel and finally influence ion channel opening/closing. With GlyR β mutations it should also be noted that the association with gephyrin might be changed even though the mutation is not localized in the GlyR β ICD harboring the gephyrin binding site. It would be of interest to analyze GlyR β mutants in a neuronal context to determine possible changes in synaptic localization.

Besides the large extracellular N-terminus, GlyRs harbor a short extracellular loop between transmembrane domains 2–3 (TM2-3), and an extracellular C-terminus. All three ECD regions form together the GlyR ECD. The very short C-terminal region lacks GlyR α1 or β mutations associated with hyperekplexia. In contrast, the extracellular loop between TM2-3 is highly susceptible for mutations in the GlyR α1 subunit generating a startle phenotype. K276E/Q, Y279C/S/X, and V280M have been identified in this extracellular loop (**Figure 3**) (Shiang et al., 1995; Elmslie et al., 1996; Bode and Lynch, 2013). All three mutations are dominant. K276 and Y279 were found in different families (Shiang et al., 1995; Elmslie et al., 1996). The *in vitro* analysis showed that the functionality of these residues is highly impaired but transport to and integration into the cellular membrane is not affected. Glycine sensitivity is significantly decreased for both mutants K276 and Y279. In addition, K276E elicits a reduced open probability (Lynch et al., 1997; Lewis et al., 1998). Using a combined approach of electrophysiological recordings to study ion channel physiology coupled to readout of changes in fluorescence, it was hypothesized that the TM2-3 loop is an important gating element of GlyRs (Wang and Lynch, 2011). These results were further confirmed by the cryo-EM structure of GlyR α1 and the GlyR α3 determined by X-ray crystallography (Du et al., 2015; Huang et al., 2015). The extracellular TM2-3 loop interacts with residues from the large N-terminal GlyR domain and is involved in structural rearrangements following ligand binding enabling finally signal transduction resulting in ion channel pore opening (Du et al., 2015).

### Mutations in the GlyR TMD

#### TM1

Except Q226E and W239C, mutations that have been found in TM1 associated with human hyperekplexia are recessively inherited, e.g., Y228C, P230S, S231R/N, and I244N. P230S and S231N were identified as compound heterozygous mutations together with R65W and S296X (Bode and Lynch, 2013; Chung et al., 2013). The dominant mutation Q226E does not impair trafficking to the cell surface which is in line with the general assumption that dominant mutations affect ion channel function but not receptor trafficking. Residue Q226 is localized near the top of TM1 and thus closely opposed to R271 at the top of TM2 of the neighboring subunit. Q226E showed spontaneous open channels and a slight rightward shift of the glycine EC_50_. The observed increased glycine EC_50_ of Q226E did not change in the presence of GlyR α1 wild type or wild type subunits α1 and β. The exchange of glutamine into the negatively charged glutamate is proposed to activate the channel by electrostatic attraction of the resulting E226 to R271 in TM2 (Bode and Lynch, 2013). Spontaneous openings have also been recognized for the startle disease mutant in the TM2-3 loop, e.g., K276E. From structural data it is hypothesized that mutations in M1 as well as the TM2-3 loop disrupt the van der Waals contacts of residue S278 with residues in the pre-M1/M1 region and interactions with the β8-β9 loop tip (N-cap). The disruption of van der Waals interactions is thought to underlie the spontaneous channel openings observed for startle disase mutants in M1 and the TM2-3 loop (Du et al., 2015).

Single analysis of P230S revealed functional homomeric channels with fast desensitizing currents. The introduction of a serine instead of the proline introduces most probably major conformational changes by a profound kink at the extracellular site of TM1 (Bode and Lynch, 2013). Impaired receptor biogenesis has been further described for S231R and I244N (Villmann et al., 2009b). In contrast to I244N, the surface expression of S231R is reduced to 50%, however the number of expressed GlyRs at the cellular surface is sufficient to form functional ion channels with reduced glycinergic maximal current amplitudes (S231R showed 50% reduced I_max_ values compared to GlyR α1 when expressed as homomers as well as in heteromeric expression with GlyR β; I244N showed 10-fold reduction of I_max_ when expressed as homomeric channels, and threefold reduction when coexpressed with GlyR β). Therefore, the coexpression with the GlyR β subunit such as the *in vivo* complex formation leads to less severe functional effects compared to GlyRs composed of five mutated α1 subunits. Protein stability of S231R and I244N was significantly reduced (9 h compared to GlyR α1 wild type with 24 h before protein is nicked into N- and C-terminal fragments). These data suggest that mutants result in instable protein and probably higher protein-turnover rates. A block of the proteasomal pathway demonstrated that S231R and I244N accumulate in the cell arguing that proteasomal degradation is one of the pathways used for mutated GlyR protein degradation (**Figure 4C**) (Villmann et al., 2009b).

Within the GlyR β subunit sequence of TM1 a deletion of serine 262 has been identified (Chung et al., 2013). The mutant ΔS262 shows a profound reduction of surface expression in line with reduced chloride influx while glycine sensitivity was almost unchanged (Chung et al., 2013).

#### TM2

TM2 is described as a hotspot region within the GlyR α1 where most dominant ion channel mutations are localized (V260M, T265I, Q266H, S267N, S270T, R271Q/L/F). The TM2 of five subunits form the inner wall of the ion channel pore und thus the ion permeation pathway. The constriction of the ion channel pore differs between open, closed, and desensitized stages but also between the upper, central, and lower part of the pore (Du et al., 2015). During ion channel opening/closing this domain undergoes large clockwise or anticlockwise rotations. It is thus not surprising that changes in the side chain volume or charge of TM2 residues impair functionality of the GlyR.

For dominant mutants a reduction in GlyR trafficking has not been observed indicating that structural changes not unnecessarily lead to protein misfolding and trafficking defects. The first GlyR α1 mutant identified in a family with hyperekplexia was R271Q localized in the upper half of the ion channel pore (Shiang et al., 1993). Although this mutant is not directly involved in binding of β-alanine and taurine, it changes both substances from partial agonists to antagonists, involved in gating (Du et al., 2015). The two mutants R252C/H and G254D localized at the inner mouth of the ion channel are recessive. Both result in reduced GlyR α1 trafficking and non-functionality of R252C/H and G254D, even with presence of the GlyR β subunit (Chung et al., 2010).

TM2 of the GlyR β subunit carries two missense mutations R276X, L285R. James et al. (2013) characterized this double mutant with reduced cellular surface expression. Functional analysis of L285R was done coupling electrophysiological readouts to changes in fluorescence. The amino acid position 285 is located in TM2 with the positively charged side chain of the arginine mutant pointing toward the center of the pore. The configurations α1β^*L*285*R*^ and α1ββ^*L*285*R*^ showed an increase in fluorescent quench relative to heteromeric α1β GlyRs in the absence of glycine suggestive for spontaneous activity of this GlyR β mutant (James et al., 2013). These data are in line with artificial mutations at the 9′ position in GABA_A/C_R subunits resulting in spontaneous channel openings (Chang and Weiss, 1998, 1999).

#### TM3

Two recessive mutations have been identified in TM3 of the GlyR α1 subunit. L291P introduces a proline within the α-helix of TM3. Prolines introduce destabilizing kinks into α-helices due to the ring formation by its side chain, which prevents its amino group from formation of the usual H-bond. Hence, the conformational change of the α-helix of TM3 might underlie the lack of function. A switch in the positioning of TM3 in relation to TM1 and TM4 is also a possible mechanism to explain lack of function for this mutant. In contrast, S296X introduces a stop codon and results in a TM3 truncation of GlyR1 α1. Both mutants, L291P and S296X, were unable to generate functional channels when expressed in a homomeric configuration *in vitro* following transient transfection into HEK293 cells (Bode and Lynch, 2013; Chung et al., 2013). For both mutants, coexpression of L291P and S296X, together with either α1 or α1β, resulted in functional GlyRs with reduced dominant-negative effects on the overall Cl^-^ current density (Bellini et al., 2007; Bode and Lynch, 2013). The reduced glycine efficacy for L291P might result from reduced cell surface expression. Furthermore, the glycine potency for L291P was also reduced threefold (Bode and Lynch, 2013).

The mutation S321F found in TM3 of the *GLRB* gene has not yet been investigated, neither for trafficking nor for function (Lee et al., 2013). In contrast, W310C showed reduced surface expression accompanied by reduced Cl^-^ currents. The exchange of the aromatic side chain of tryptophan 310 into a cysteine disrupts the contribution of the tryptophan to the hydrophobic stack of TMs 1–3.

#### TM4

The analysis of patients suffering from hyperekplexia revealed one dominant (R414H) and two recessive mutations in TM4 of GlyR α1 (R392H and W407R) (Vergouwe et al., 1999; Bode and Lynch, 2013; Schaefer et al., 2015). Interestingly, the expression level as well as the glycine EC_50_ of the dominant mutation R414 was indistinguishable from GlyR α1 wild type leaving the pathology of this mutant so far unclear (Bode and Lynch, 2013). The mutation R392H is localized at the intracellular site of TM4. This mutant showed a reduced surface expression and residual Cl^-^ currents. Coexpression with GlyR β rescued the observed reduced maximal currents but still α1^*R*392*H*^/β exhibited an eightfold decrease in glycine EC_50_ (Chung et al., 2013). Again, the severity of the functional deficit is less pronounced in the heteromeric GlyR complex. Originally R392H was identified in a patient compound heterozygous to R252H localized at the inner entrance of the ion channel pore (Vergouwe et al., 1999). Coexpression of R392H with R252H led to a dominant-negative effect of R252H over R392H with non-functionality of the GlyR similar to homomeric R252H channels. Coexpression of both mutated GlyR α1 subunits R252H and R392H readjusted the GlyR α1 *in vivo* situation in compound heterozygous patients suffering from hyperekplexia (Villmann et al., 2009b). In addition, both mutants R252H and R392H exhibited low protein stability. Protein degradation started 1 h after synthesis for R392H whereas the wild type protein was stable for 24 h (Villmann et al., 2009b). It was also hypothesized that both affected arginine residues localized at the inner site of either TM2 and TM4 might disrupt topogenic signals important for correct integration of TMs into the cellular membrane.

The recessive GlyR α1 mutant W407R was detected in a patient together with D70N. The mutant was almost absent from the cell surface and neither coexpression with D70N nor coexpression with the β subunit rescued surface expression or functionality (Schaefer et al., 2015). Mutating aromatic residues of TM4 and in other TM regions including residue W407 demonstrated that the lack of the aromatic residue W407 contributes to the disruption of the aromatic ring network important for intramembrane π–π interactions essential for pentamerization of the GlyR complex (Haeger et al., 2010). Thus, both studies provide evidence that W407 is a key residue for GlyR pentamerization.

Data on the recessive mutation Y470C identified in *GLRB* follow the same line of research. Y470C is less expressed at the cell surface accompanied by reduced chloride ion influx, but displays no changes in glycine potency (Chung et al., 2013).

In summary, mutations affecting GlyR biogenesis have mostly been characterized by decreased surface expression. There are single examples where a precise picture on trafficking through cellular compartments was provided or degradation pathways have been elucidated. Still, a more detailed picture on GlyR trafficking under disease conditions would extend and clarify our current understanding of the pathology in startle disease.

### Mutations in the GlyR α1 ICD

#### TM1-2 Loop

The intracellular TM1-2 loop is short and consists of 10 amino acids. The only known mutation associated with hyperekplexia is GlyR α1 P250T. P250T is a dominant mutant which reduces glycine efficacy and potency. Whether the observed reduced glycine efficacy is due to reduced protein synthesis and trafficking to the cellular membrane has not yet been examined. The most significant functional change obtained for P250T is the dramatically accelerated desensitization of this mutant, arguing that fast ion channel closure underlies the pathology of impaired GlyR signaling (Saul et al., 1999; Breitinger et al., 2001). A study on mixed coexpressions in defined ratios of GlyR α1 P250T (desensitizing) and wild type (non-desensitizing) subunits investigated the dominant negative effect of this mutant. Mixed desensitization time constants were observed suggesting that in contrast to the dominant effect of P250T in the human patient, in the recombinant HEK293 overexpression system the wild type GlyR α1 subunits dominated (Breitinger and Becker, 2002).

#### TM3-4 Loop

Except for one dominant GlyR α1 mutation, GlyR mutations found in the large intracellular loop are recessive. Some of the recessive GlyR mutations were originally identified combined with other recessive mutations elsewhere in the protein sequence leading to compound heterozygosity in the human patient. Both affected alleles contribute to the hyperekplexia phenotype.

The GlyR α1 mutation G342D is one of the exceptions resulting in functional chloride channels indistinguishable from GlyR α1 wild type function (Rees et al., 2001). Very recently, the mutation A384P was not shown to have trafficking defects but is characterized by accelerated desensitization kinetics comparable to the fast desensitization of P250T localized in the intracellular TM1-2 loop (Wang et al., 2018). In contrast to the dominant mutant P250T, A384P was identified in a compound heterozygous patient together with another TM3-4 loop mutation R392H (Mine et al., 2015). R392H elicits residual GlyR function, which increased upon coexpression with the GlyR β subunit (Chung et al., 2010). The R392H mutation has not been explored together with A384P *in vitro*.

Another missense mutation in the TM3-4 loop is GlyR α1 D388A. No surface expression for D388A was observed when expressed as α1 homomers. Coexpressions of D388A together with either wild type α1 or α1β generated functional ion channels with distinct ion channel properties and different from α1 wild type homopentamers or α1β heteropentamers (3–4 fold increase in glycine EC_50_ compared to α1 or α1β wild type) (Bode and Lynch, 2013).

Within the TM3-4 loop nonsense mutations are common with R316X and E375X in the GlyR α1 subunit and R450X in the GlyR β subunit (Bode and Lynch, 2013; Chung et al., 2013). These mutants are expressed *in vitro* and result in reduced surface expressions. Truncated GlyR α1 or GlyR β subunits form no functional ion channels unless coexpressed with the wild type GlyR α1 or α1β subunits (Bode et al., 2013; Schaefer et al., 2015). If these truncated GlyR proteins are expressed *in vivo* is questionable. The GlyR mouse mutant *oscillator* harboring a deletion of 7 bp in the TM3-4 loop does also result in a premature STOP codon (Buckwalter et al., 1994). Protein expression of truncated GlyR α1 *in vivo* has never been observed. Interestingly, *in vitro* the coexpression of the truncated protein together with the missing GlyR α1 part encoded on a different plasmid was not only able to restore protein trafficking to the cellular surface but also restored ion channel functionality (Villmann et al., 2009a). Similarly, Haeger et al. (2010), showed that GlyR domains are able to complement each other to functional receptors. When the human mutant R316X was coexpressed with the lacking C-terminal domain (iD-TM4-C), strychnine binding was rescued revealing a similar binding affinity of R316X coexpressed with the C-terminal domain compared to wild type GlyR α1. *B*_max_ was reduced to 30%, which is consistent with decreased levels of expressed receptors at the PM. In functional analysis using whole cell recordings from cotransfected R316X together with the lacking C-terminal domain in HEK293 cells, again 30% rescue of I_max_ was found (Schaefer et al., 2015). A similar reduction of I_max_ and a fourfold increase in glycine EC_50_ has been observed when R316X was coexpressed with α1 or α1β wild type (Bode and Lynch, 2013).

As type of interactions between N- and C-terminal domains of the GlyR, π–π interactions at the aromatic interfaces of TM1, TM3, and TM4 and tight intersubunit interactions between the ECD are discussed. When the GlyR protein is truncated as observed for human GlyR α1 variants associated with startle disease or in the mouse mutant *oscillator*, tight intersubunit interactions between the ECD are most probably unchanged. The lack of TM4 however hinders pentamerization but not randomized oligomerization of truncated GlyRs in the ER by an impairment of the aromatic network between TM interfaces. The intramembrane network is thus a key structural element for GlyR assembly and transport of correctly pentamerized GlyR complexes to the cellular surface.

## Enhanced GlyR Internalization Upon GlyR Autoantibody Binding in Patients With SPS

**FIGURE 5:**
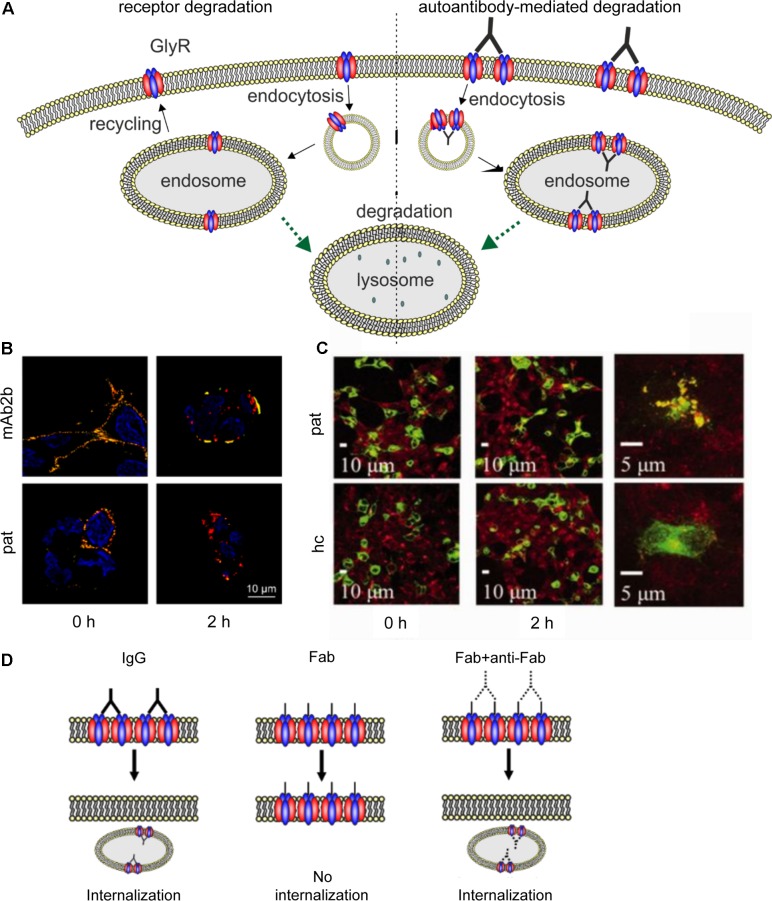
Native GlyR internalization compared to autoantibody-mediated GlyR internalization. **(A)** Under native conditions, ubiquitination of cell surface GlyRs initiates receptor degradation by endocytosis followed by lysosomal or lysosomal-like vacuolar degradation (left). Autoantibodies crosslink GlyRs, thus inducing internalization and degradation by endosomal and lysosomal pathways (right). **(B)** GlyR α1-transfected HEK293 cells were incubated with monoclonal GlyR α1-specific antibody mAb2b or patient serum (pat) and internalization was induced by incubation at 37°C for 0 or 2 h. Patient serum as well as mAb2b were able to induce internalization at 2 h. Red = internalized GlyRs; yellow = membrane-integrated GlyRs; blue = DAPI staining. **(C)** GlyR α1-EGFP expressing HEK293 cells were incubated with patient serum for 0 or 2 h at 37°C to induce internalization and stained with late endosomal marker LAMP2. Colocalization of both signals is higher in cells incubated with patient serum than with healthy control (hc). Green = GlyR α1-EGFP signal; red = LAMP2 signal (taken with permission from Carvajal-Gonzalez et al., 2014). **(D)** IgG binding to receptors is able to induce internalization (left), whereas Fab fragments alone cannot elicit receptor internalization (middle). Internalization can re-occur, when Fab fragments and anti-Fab antibodies are incubated together (right), indicating that crosslinking of receptors is required for internalization.

Patients carrying GlyR autoantibodies suffer from SPS. For autoantibody-mediated diseases, receptor crosslinking has been suggested to underlie disease pathology. Receptor crosslinking by autoantibodies was first described in Myasthenia gravis, an autoimmune disease with crosslinking of nicotinic acetylcholine receptors which also belong to the CLR family such as the GlyR (**Figure 5A**) (Drachman et al., 1978; Hughes et al., 2004). Since these first findings, three different pathomechanisms for autoantibody-mediated diseases have been suggested: (i) antigenic stimulation of receptor internalization subsequent to receptor crosslinking (**Figures 5A,B**), (ii) complement activation following autoantibody binding to target receptors, and (iii) blocking of receptor function by binding of autoantibodies to the target receptor.

### Antigenic Stimulation of Receptor Internalization Subsequent to Receptor Crosslinking

Carvajal-Gonzalez et al. (2014) provided the first data concerning the pathomechanisms of GlyR autoantibodies using cellular approaches. GlyR autoantibody binding was followed over a period of 5 min to 16 h using immunocytochemical staining of GlyR α1 expressing HEK293 cells that were incubated at 4 or 37°C. Incubation at 37°C reduced the number of receptors with surface-bound autoantibodies whereas at 4°C no change in surface receptor numbers has been observed. Receptor internalization following incubation with the autoantibodies for 2 h at 37°C led to a reduction of remaining surface-bound antibodies to about 7% (compared to about 52% in healthy control) which was further decreased after 16 h. The internalized GlyRs colocalized with the late endosomal marker LAMP2 (**Figure 5C**). Further investigation of the autoantibody targeted GlyR subsequent to the endosome has not been shown. Such studies would help to understand changes in the life cycle of GlyRs under disease conditions. It was proposed that autoantibodies divalently bind to GlyRs and thus induce receptor internalization (Carvajal-Gonzalez et al., 2014). Although the whole study investigated serum from a number of patients, the experimental series on receptor internalization was done with the serum of one patient only. Since the disease pattern of SPS patients carrying GlyR autoantibodies differs, the underlying pathomechanisms might also vary between patient samples. This aspect has not been investigated yet.

Further insights into the pathology of autoantibodies against receptor proteins can be obtained from autoantibodies targeting other ligand-gated ion channels. Receptor internalization as a consequence of autoantibody binding could also be determined for autoantibodies targeting the excitatory ligand-gated NMDA receptors (Dalmau et al., 2008; Hughes et al., 2010; Moscato et al., 2014; Planagumà et al., 2015; Castillo-Gomez et al., 2017; Pan et al., 2018). Incubation of hippocampal neurons with patient autoantibodies between 15 min and 48 h or even up to 7 days could significantly reduce NMDA surface cluster densities, which was titer-dependent (Dalmau et al., 2008; Hughes et al., 2010; Moscato et al., 2014). Although NMDA receptor clusters were reduced by autoantibody binding, the number of excitatory synapses, neuronal morphology or viability were unaltered (Hughes et al., 2010). As the underlying pathomechanism, antibody-mediated crosslinking of receptors as a prerequisite to receptor internalization was proposed. Compared to GlyR autoantibodies, for NMDA receptor autoantibodies this mechanism was experimentally proven by generating Fab fragments of autoantibodies which alone were not able to induce internalization (**Figure 5D**) (Hughes et al., 2010; Moscato et al., 2014). Additionally, incubation with Fab fragments and anti-Fab secondary antibodies, which link two Fab fragments similar to unmodified patient autoantibody, significantly reduced the NMDA receptor cluster density compared to control IgG (Hughes et al., 2010). Moreover, significant decrease in NMDA receptor cluster density could also be detected by injecting autoantibodies directly into the hippocampus of rats *in vivo* as well as by immunostaining of human hippocampus (Hughes et al., 2010). So far, *in vivo* studies of GlyR autoantibodies as well as long-term effects of GlyR autoantibodies have not been shown.

### Complement Activation Following Autoantibody Binding to Target Receptors

The complement system is an important mediator between innate and acquired immunity and improves removal of foreign or damaged cells by binding of immune complexes and interacting with IgG and IgM antibodies (Davies et al., 1994; Nesargikar et al., 2012). For GlyR and NMDA receptor autoantibodies it was demonstrated that both were subclassified as predominantly IgG1 and IgG3 (Dalmau et al., 2008; Tuzun et al., 2009; Hughes et al., 2010; Carvajal-Gonzalez et al., 2014; Balint and Bhatia, 2016; Chefdeville et al., 2016). Both subclasses, IgG1 and IgG3, are able to activate the complement system. Complement activation has been shown by accumulation of the C3b component of the complement cascade (Carvajal-Gonzalez et al., 2014). The activation of the complement system might thus also contribute to GlyR autoantibody pathology. So far however, GlyR crosslinking by GlyR autoantibodies leading to enhanced receptor internalization is favored as the major pathomechanism in SPS (Carvajal-Gonzalez et al., 2014; Balint and Bhatia, 2016; Chefdeville et al., 2016). Although NMDA receptor autoantibodies are of the same IgG1 or IgG3 class, they do not cause complement-mediated damage (Dalmau et al., 2007, 2008; Tuzun et al., 2009; Hughes et al., 2010; Irani et al., 2010; Martinez-Hernandez et al., 2011; Planagumà et al., 2015). In patients with anti-NMDA receptor encephalitis that additionally suffer from teratoma cancer, complement immunoreactivity was present in 81% of teratomas but not in autopsied brain regions (Tuzun et al., 2009; Martinez-Hernandez et al., 2011). In contrast, Irani et al. (2010) could show that NMDA receptor-transfected HEK293 cells were able to deposit complement C3b. Thus, these discrepancies reveal that one might be careful with a direct comparison between data obtained from *in vitro* overexpression and *in vivo* data. However, variability in the clinical phenotype might also underlie such contradictory results.

### Blocking of Receptor Function by Binding of Autoantibodies to Its Target

Beyond receptor internalization, autoantibody binding to surface receptors could also affect receptor function. For GlyR autoantibodies, this issue has not been examined yet. Again, we have learned from other types of ligand-gated ion channels such as the excitatory NMDA receptors that glutamate-evoked currents were significantly reduced when cells were treated 6 min with patient sera containing NMDA receptor autoantibodies (Castillo-Gomez et al., 2017). In contrast, incubation of hippocampal neurons with patient autoantibodies for 30 min exhibited no significant differences of mEPSCs compared to control in whole-cell patch-clamp experiments (Moscato et al., 2014). These data propose that the pathomechanism of NMDA receptor autoantibodies most probably results from both enhanced receptor internalization following receptor crosslinking but also from altered receptor function.

Taken together, GlyR internalization by autoantibody binding and degradation in endosomes as pathomechanism in SPS patients was proven. Further aspects like crosslinking of the receptors by autoantibodies and/or possible alterations in receptor function, which have been shown in patients carrying NMDA receptor autoantibodies, still needs to be explored for GlyR autoantibodies.

## Mouse Models With Defective Trafficking of GlyRs Associated With Disease

Six startle disease mouse models are currently available, all of which are inherited recessively. *Spasmodic* (*spd*), *oscillator* (*spd^ot^*), *spastic* (*spa*), *shaky* (*sh*) and *cincinnati* occurred spontaneously while *Nmf11* was chemically induced (Lane et al., 1987; Becker, 1990; Buckwalter et al., 1994; Holland et al., 2006; Traka et al., 2006; Schaefer et al., 2017). These mouse lines serve as model systems for the human neuromotor disorder startle disease and share phenotypic symptoms with the human situation. In mice, symptoms start at the age of 2–3 weeks after birth with increased startle reflex, tremor, impaired righting reflex, and muscle spasms. *Spasmodic* is the only mutant with a normal lifespan, all other mutations result in premature death after 3–6 weeks (Schaefer et al., 2012). Furthermore, knock-in mice are available carrying startle disease mutations. Studies of the ethanol-resistant GlyR α1 mutations Q266I and M287L in knock-in mice revealed typical startle disease-like symptoms and differences in alcohol-related behavior, e.g., ethanol consumption, ethanol-stimulated startle response, and recovery from rotarod ataxia (Blednov et al., 2012; Borghese et al., 2012). Besides the startle disease-like phenotype, most of these mouse models have been characterized at the mRNA, DNA, and protein level.

The underlying mutations in the spontaneous mouse models affect either the *Glra1* or the *Glrb* gene. *Spasmodic* results from the GlyR α1 mutation A52S accompanied by decreased glycine affinity (Ryan et al., 1994; Saul et al., 1994). *Oscillator* mice harbor a 2 bp microinsertion and a 9 bp microdeletion in *Glra1*, resulting in two non-functional GlyR α1 splice variants, both lacking TM4 (Buckwalter et al., 1994; Kling et al., 1997). Insertions of a repetitive LINE1 element into *Glrb* intron 6 and a SNP in exon 6 of *spastic* mice lead to a 90% reduction of GlyR β mRNA level and largely decreased numbers of heteromeric synaptic GlyRs (Kingsmore et al., 1994; Mulhardt et al., 1994; Becker et al., 2012). S*haky* carries a missense mutation in *Glra1* exon 6 (Q177K), resulting in decreased receptor function and synaptic clustering (Schaefer et al., 2017). In *cincinnati* mice, duplication of *Glra1* exon 5 generates a premature stop codon (F159X) and non-functional GlyRs (Holland et al., 2006). The *Nmf11* mutation N46K, induced by the chemical mutagen ENU, reduces glycine potency as a consequence of rapid receptor deactivation (Traka et al., 2006; Wilkins et al., 2016). Further studies on some of these mouse models concentrated on functional aspects to explain disease progression (Graham et al., 2003, 2006). Studies on GlyR trafficking and protein levels are rare.

Glycine receptor trafficking defects were observed in *shaky* and *oscillator.* Synaptic localization of α1-containing GlyRs in homozygous *shaky* mice *in vivo* was significantly decreased, while GlyR α1 protein expression was enhanced in spinal cord and significantly increased in brain stem compared to wild type control mice. Expression of the scaffold protein gephyrin was increased concomitantly (Schaefer et al., 2017). Functional analysis by electrophysiological recordings from brain stem slices revealed significantly reduced current amplitudes, lower IPSC frequencies, and decreased desensitization decay time constants. Schaefer and colleagues argue that the impaired function might result in increased turnover of synaptic receptors via endocytosis and an enhanced expression as an attempted compensation. Furthermore, the mutation may alter conformational changes transduced to the GlyR β TM3-4 domain, which harbors the gephyrin binding side, and therefore disrupts synaptic anchoring (Schaefer et al., 2017). Hence, these data show that upregulation of the expression level of the mutated GlyR α1 and gephyrin in *shaky* mice are not sufficient to rescue synaptic localization of GlyR-gephyrin complexes. As a mechanism of inhibitory compensation, one might think of enhanced synaptic GABA_A_ receptor formation due to increased gephyrin expression. Despite the fact that GABA_A_ receptor expression has not been investigated by Schaefer and colleagues, gabaergic compensation for the largely decreased glycinergic inhibition can be excluded as homozygous *shaky* mice die 4–6 weeks after birth.

The *shaky* mutation Q177K is located in the β8–β9 loop of the large ECD of the GlyR α1 subunit. This loop forms, together with neighboring loops, the ligand-binding pocket. Conformational changes of these loops induced by ligand-binding help to transfer the closed receptor into the open state and back (Du et al., 2015; Huang et al., 2015). The β8-β9 loop further contributes to a hydrogen bond network important for channel opening (Padgett and Lummis, 2008; Nys et al., 2013). Modeling revealed that the *shaky* mutation disrupts the hydrogen bond network around residue 177 and thus impairs binding of the agonist to the ligand-binding pocket. *In vitro* studies have shown that changing the amino acid glutamine at position 177 into amino acids with different charge, longer/shorter side chains, or increased/decreased volume results in increased glycine EC_50_ values and decreased potency of the partial agonists β-alanine and taurine (Janzen et al., 2017). Furthermore, replacing the neutral glutamine Q177 with positively charged lysine or arginine, as well as neutral amino acids glycine, alanine, cysteine, and tryptophan, significantly reduced GlyR α1 surface expression in transfected HEK293 cells independent of the GlyR β expression. Whole-cell expression was however unaffected for all GlyR α1^Q177^ variants. These data on GlyR biogenesis of GlyR α1^Q177^ variants provide hints for a possible maturation deficit that may lead to ER retention *in vitro*, as shown before for other recessive GlyR α1 mutations (Schaefer et al., 2015; Janzen et al., 2017).

A more severe trafficking defect has been observed in *oscillator* mice. GlyR α1*^spd-ot^* are non-functional due to lack of GlyR α1 in spinal cord membranes of homozygous *oscillator* mice (Kling et al., 1997; Schaefer et al., 2017). Furthermore, Kling et al. (1997) observed a dramatic reduction in gephyrin membrane levels in the spinal cord of *oscillator* mice. The reduction of both proteins, GlyR α1 and gephyrin, is in line with the current GlyR trafficking model of gephyrin binding to fully assembled GlyRs in the ER of neurons and forward trafficking to the membrane only following the successful formation of this protein complex (Hanus et al., 2004). The *oscillator* phenotype is lethal 3 weeks after birth, proposing that lack of glycinergic inhibition cannot be compensated by the closely related and also gephyrin anchored GABA_A_ receptors present at the same synapses. It seems that no gabaergic compensation exists for the reduced or lack of glycinergic inhibition observed in mice with startle disease. With this, mechanisms of compensation differ between mice and humans. Patients suffering from startle disease are treated with diazepam, a positive allosteric modulator of the GABA_A_ receptor. Since the treatment works at the symptomatic level in human patients, it is suggested that lack of glycinergic inhibition is rescued by an increase of gabaergic function upon diazepam treatment. The molecular mechanism of diazepam compensation is however so far not understood.

As pointed out before, alternative splicing using two splice acceptor sites in *Glra1* exon 9 generates two GlyR α1 variants as a consequence of the *oscillator* mutation (Malosio et al., 1991). This results in an elongated variant (spd^*ot*^-elg) with 150 C-terminal missense residues and a truncated variant (spd^*ot*^-trc), characterized by a premature STOP codon, missing most of the TM3-4 loop, TM4, and the extracellular C-terminal domain (Villmann et al., 2009a). In both variants, the basic motif ^318^RRKRRH^323^, located at the beginning of the TM3-4 loop, is mutated. These residues have been shown to be essential for forward GlyR trafficking to the PM. Neutralization of one or more positive residues partially results in incorrectly folded GlyR α1 subunits that are withheld in the ER (Sadtler et al., 2003). Coexpression of spd^*ot*^-trc together with a tail construct, encoding for the missing C-terminal part and promoting receptor pentamerization, enabled transport of functional ion channels to the membrane albeit with reduced protein levels. Introduction of the basic motif into spd^*ot*^-trc increased surface expression levels of both independent domains, confirming the importance of these positively charged residues for GlyR trafficking (Villmann et al., 2009a).

The pre-synaptic form of startle disease is caused by mutations in GlyT2. A mouse line lacking functional GlyT2 was first described by Gomeza et al. (2003). GlyT2^-/-^ knockout mice exhibit severe muscle spasms and die during the second week after birth (Gomeza et al., 2003). Deletion of GlyT2 does not affect expression or localization of GlyRs and other synaptic proteins, however, GlyR-mediated mIPSCs are reduced (Gomeza et al., 2003). Another mouse mutant displaying similar symptoms was described by Bogdanik et al. (2012). Insertion of a MusD retrotransposon into the GlyT2 gene *Slc6a5* abolishes protein expression in homozygous mice, most likely by disturbing translation or destabilizing *Slc6a5* transcripts.

Trafficking defects have only been described for GlyT2 missense mutations found originally in human patients. The mutation Y705C impairs transport of GlyT2 to the cell surface due to formation of an aberrant disulfide bond (Gimenez et al., 2012), while S512R leads to enhanced binding of misfolded receptors to calnexin, altered interaction with COPII, and formation of oligomers containing both mutant and wild type GlyT2s that remain in the ER (Arribas-Gonzalez et al., 2015).

Gephyrin knock-out mice display a phenotype resembling startle disease and result in reduced synaptic clustering of GlyRs. In addition to enabling GlyR or GABA_A_ receptor clustering at post-synaptic sites, gephyrin is also involved in molybdenum cofactor synthesis. Thus, gephyrin knockout mice result in molybdenum cofactor deficiency attributed to impaired molybdenum cofactor synthesis (Feng et al., 1998).

GABA_A_ receptors are, like GlyRs, part of the superfamily of CLRs and mediate fast synaptic inhibition in the brain (Sieghart, 2006). Mutations of GABA_A_Rs are associated with epilepsy, anxiety disorders, and schizophrenia (Braat and Kooy, 2015). Similar to the GlyR *oscillator* mutation, a truncated GABA_A_R receptor mutant was identified in a patient with severe myoclonic epilepsy in infancy. The mutation affects the GABA_A_ receptor subunit γ2^Q390X^ (formerly Q351X). An *in vitro* analysis revealed ER retention of this γ2 subunit variant (Harkin et al., 2002). Similar to the role of GlyR β and gephyrin in the GlyR, the GABA_A_ γ2 subunit and gephyrin are essential for receptor trafficking and clustering at the synapse (Essrich et al., 1998; Schweizer et al., 2003). The role of GABA_A_ γ2 subunit is however not equivalent to GlyR β, since no interaction of GABA_A_ γ2 with gephyrin is yet shown. Recently, a novel mechanism for γ2-dependent and gephyrin-independent synaptic localization of GABA_A_R was described. GARLH/LHFPL4 was identified as a putative auxiliary subunit of GABA_A_Rs. This protein is enriched in inhibitory synapses and forms a tripartite complex with neuroligin-2 and γ2-containing GABA_A_Rs (Davenport et al., 2017; Yamasaki et al., 2017). Thus, it can be assumed that lack of interaction with the auxiliary subunit due to the GABA_A_R truncation in the γ2 subunit (γ2^Q390X^) might underlie the pathology in severe myoclonic epilepsy in infancy.

*In vitro*, the truncated mutant GABA_A_ γ2^Q390X^ revealed oligomerization with wild type γ2 subunits and trapping of the resulting receptors in the ER (Kang et al., 2009). The *Gabrg2*^+/Q390X^ knock-in mouse, a new mouse model for human genetic epileptic encephalopathy, identified accumulation and aggregation of mutant γ2 subunits in neurons as a prerequisite for neurodegenerative processes, previously not associated with genetic epilepsies (Kang et al., 2015).

So far, hyperekplexia mouse models have been used to elucidate GlyR function and how functional defects cause the disease. Additional trafficking defects may however exist that would explain the observed functional effects. Future studies on GlyR trafficking mechanisms in startle disease mouse models may help to get a better understanding on impaired GlyR transport and signaling under disease conditions. Hence, the known GlyR mouse mutants offer excellent research tools for the investigation of mutant GlyR trafficking in the *in vivo* situation.

## Outlook

This review summarizes the trafficking pathways of GlyRs under native and disease conditions. Most data have been obtained from *in vitro* studies. So far, reduced surface expression and/or reduced functionality and less synaptic strength are discussed as mechanism leading to reduced fast inhibitory neurotransmission in the adult organism under disease conditions.

Although startle disease patients and SPS patients share some phenotypic similarities, the underlying mechanisms of targeted GlyR are distinct. In startle disease, mutations in GlyRs affect GlyR structure and hence correct assembly, oligomerization/pentamerization and forward trafficking to the cellular surface and finally result in inhibitory malfunction. In SPS, GlyR trafficking to the cellular PM is unaffected but autoantibody-mediated crosslinking of the targeted GlyRs enhance receptor internalization. The GlyR ECD is suggested as binding site for autoantibodies but the correct epitope(s) of GlyR autoantibodies is/are still unknown. It is still unclear if GlyR autoantibodies are the real cause for the SPS pathology. Furthermore, it would be interesting to investigate long-term exposure of GlyR autoantibodies which would reflect the situation in the patient to see how or if GlyR homeostasis may counteract enhanced GlyR internalization in a longer time window. Furthermore, it is worth investigating if functional differences exist following autoantibody binding to its target receptor.

To get a deeper insight into the *in vivo* situation under disease conditions such as startle disease, mouse models helped to increase our knowledge at the molecular mechanisms. Especially, *shaky* refers to a good GlyR α1 model with its reduced synaptic GlyRs, *ocillator* serves as a tool to investigate receptor truncations, the *spastic* mutation leads to less than 15% of GlyR β. Modern techniques such as high-resolution microscopy to precisely characterize the structure of synapses and changes in the diffusive behavior of mutated GlyRs are available and have been used to study receptor trafficking. A comprehensive investigation of the trafficking pathways in GlyR mutant mice as well as investigating the transport of GlyRs bound to autoantibodies subsequent from the endosome will uncover similarities and discrepancies of GlyR biogenesis, recycling, and degradation under different disease conditions and thus significantly extend our current understanding of glycinergic dysfunction in the central nervous system.

## Ethics Statement

Experiments were approved by a positive votum of the ethics committee of the Medical Faculty of the University of Würzburg concerning an experimental series to “Autoantibodies and glycinergic dysfunction – Pathophysiology of associated motor disorders”.

## Author Contributions

NS, VR, and CV performed the immunocytochemical staining. NS and CV conducted protein analyses from the transfected cells. NS, DJ, VR, and CV participated in the manuscript writing.

## Conflict of Interest Statement

The authors declare that the research was conducted in the absence of any commercial or financial relationships that could be construed as a potential conflict of interest.

## Abbreviations

CLR: Cys-loop receptor
ECD: extracellular domain
ER: endoplasmic reticulum
GlyR: glycine receptor
GlyT2: glycine transporter 2
ICD: intracellular domain
PM: plasma membrane
TMD: transmembrane domain
